# The Colorectal Cancer Gut Environment Regulates Activity of the Microbiome and Promotes the Multidrug Resistant Phenotype of ESKAPE and Other Pathogens

**DOI:** 10.1128/msphere.00626-22

**Published:** 2023-02-27

**Authors:** Matthew T. F. Lamaudière, Ramesh Arasaradnam, Gareth D. Weedall, Igor Y. Morozov

**Affiliations:** a Centre for Sports, Exercise and Life Sciences, Coventry University, Coventry, United Kingdom; b Divison of Biomedical Sciences, Warwick Medical School, University of Warwick, Warwick, United Kingdom; c Department of Gastroenterology, University Hospitals of Coventry and Warwickshire, NHS trust, Coventry, United Kingdom; d University of Leicester, Leicester, United Kingdom; e School of Biological and Environmental Sciences, Liverpool John Moors University, Liverpool, United Kingdom; University of Michigan-Ann Arbor

**Keywords:** colorectal cancer, metatranscriptome, gut microbiota, multidrug resistance, antibiotic resistance, ESKAPE pathogens

## Abstract

Taxonomic composition of the gut microbiota in colorectal cancer (CRC) patients is altered, a newly recognized driving force behind the disease, the activity of which has been overlooked. We conducted a pilot study on active microbial taxonomic composition in the CRC gut via metatranscriptome and 16S rRNA gene (rDNA) sequencing. We revealed sub-populations in CRC (*n* = 10) and control (*n* = 10) cohorts of over-active and dormant species, as changes in activity were often independent from abundance. Strikingly, the diseased gut significantly influenced transcription of butyrate producing bacteria, clinically relevant ESKAPE, oral, and Enterobacteriaceae pathogens. A focused analysis of antibiotic (AB) resistance genes showed that both CRC and control microbiota displayed a multidrug resistant phenotype, including ESKAPE species. However, a significant majority of AB resistance determinants of several AB families were upregulated in the CRC gut. We found that environmental gut factors regulated AB resistance gene expression *in vitro* of aerobic CRC microbiota, specifically acid, osmotic, and oxidative pressures in a predominantly health-dependent manner. This was consistent with metatranscriptome analysis of these cohorts, while osmotic and oxidative pressures induced differentially regulated responses. This work provides novel insights into the organization of active microbes in CRC, and reveals significant regulation of functionally related group activity, and unexpected microbiome-wide upregulation of AB resistance genes in response to environmental changes of the cancerous gut.

**IMPORTANCE** The human gut microbiota in colorectal cancer patients have a distinct population compared to heathy counterparts. However, the activity (gene expression) of this community has not been investigated. Following quantification of both expressed genes and gene abundance, we established that a sub-population of microbes lies dormant in the cancerous gut, while other groups, namely, clinically relevant oral and multi-drug resistant pathogens, significantly increased in activity. Targeted analysis of community-wide antibiotic resistance determinants found that their expression occurs independently of antibiotic treatment, regardless of host health. However, its expression in aerobes, *in vitro*, can be regulated by specific environmental stresses of the gut, including organic and inorganic acid pressure in a health-dependent manner. This work advances the field of microbiology in the context of disease, showing, for the first time, that colorectal cancer regulates activity of gut microorganisms and that specific gut environmental pressures can modulate their antibiotic resistance determinants expression.

## INTRODUCTION

Colorectal cancer (CRC) ranks second in cancer mortality and third in incidence, worldwide (1). The disease emerges predominantly through acquired somatic mutations over 15 to 30 years, with only around 5% of cases ascribed to inherited mutations or familial syndromes (2). Lifestyle factors, such as diet, obesity, and physical inactivity, are associated with the sporadic nature of CRC (3). It is increasingly evident that the microbial population (microbiota) of the human gut constitutes a critical determinant of gastrointestinal health, which itself is shaped directly by exogenous factors (4). Metagenome analyses have consistently shown that the composition of gut microbiota is altered during CRC, implicating a microbial role in the disease (5, 6). The gut microbiome is also a vast source of antimicrobial resistance determinants, and the activity responds to external antibiotic (AB) pressure and limits therapeutic options (7). However, whether the health status of the host and, in particular, the gut environment influences activity of the gut taxa and the regulation of AB resistance genes, has not been addressed.

It is a major achievement of recent years that 29 marker species of CRC in the gut have been identified by metagenome analyses (8). Studies have investigated roles of specific species in CRC, including enterotoxigenic Bacteroides fragilis and Escherichia coli^pks+^, and found they can induce prooncogenic phenotypes, which increases inflammation and genetic instability *in vitro* (9). However, whether activity of individual microbes within complex communities, such as the gut reflects their activity in cultures, remains to be seen. Growing lines of evidence argue that abundance of microbial taxa does not always correlate with respective transcriptome levels (10). For example, in inflammatory bowel disease (IBD), B. fragilis exhibits large disparity between prevalence and transcription, while other species like Dialister invisus are detected in metagenomic DNA but show no detectable transcription, implying they are inactive (dormant) or even dead. Therefore, an important distinction must be made between abundance and activity to avoid mis-associating species, by either under- or over-estimating their potential contribution to health and disease.

DNA-based analyses of the gut microbiome have also shown the expansion of pathogens in the CRC gut (11). Furthermore, colorectal cancer patients often require post-surgical antibiotic treatments to fight infections by E. coli, *Enterococci* spp. and “ESKAPE” pathogens, of which ~45% are AB resistant (12). ESKAPE species comprise Enterococcus faecium, Staphylococcus aureus, Klebsiella pneumoniae, Acinetobacter baumannii, Pseudomonas aeruginosa, and Enterobacter spp., and are of great clinical significance, causing nosocomial (hospital acquired) infections while possessing resistance to a broad spectrum of commonly used antibiotics. While this is consistent with the gut microbiota being a reservoir of AB resistance determinants, little is known of how the activity of AB resistance determinants is regulated in the absence of the AB pressure and by environmental factors (driven by health status) of the host gut. Therefore, understanding microbial activity and its regulation represents a significant gap in our knowledge of functional structure of the gut community in both health and disease (13).

Here, we characterized the active microbial community composition in colorectal cancer. We elucidated the levels and characteristics of microbial phylogenetic activity in 2 cohorts, non-CRC and CRC, by aligning metatranscriptomic (mRNA) and 16S rRNA gene (rDNA) sequencing for the same samples. We also analyzed expression of antibiotic resistance determinants alongside the activity of the microbiota that carry multidrug resistance determinates, including ESKAPE pathogens. We demonstrate that the activity of the human gut microbiota is regulated by the malignancy, and the CRC gut environment modulates activity of specific functionally related groups of microbes. This work brings in to focus the species that may play critical roles in either the protection of the gut and its microbial community from damage, or the promotion of malignancy.

## RESULTS

### Sequencing and community diversity metrics.

Transcriptomic sequencing of the 20 samples produced 909,748,013 read pairs, which were processed to output 693,090,228 sequences (18,510,095-38,871,022 per sample, median 21,946,018) (Data Set 1, Tab 1 and Data Set 1, Tab 2). 16S rRNA gene (rDNA) sequencing of the 20 samples produced 3,603,795 read pairs, which were processed to produce 3,303,648 sequences (Data Set 1, Tab 3). Alpha-diversity (Shannon) measured for transcriptomic and for 16S data correlated (Spearman’s ρ = 0.67, *P = *0.0017) (Fig. S1A), indicating similar signal in the data despite the different data. PERMANOVA analysis indicated the only significant grouping factor was healthy versus CRC samples (not age, sex, smoking status, or BMI) (Table S1B). This was reflected in the ordination (principal coordinate analysis [PCoA]) plots that indicated some separation between healthy and CRC samples (Fig. S1B and Fig. S1C). Alpha-diversity (Gini-Simpson) was greater in healthy samples than in CRC samples for both transcriptomic (healthy = 0.0226; CRC = 0.0170) and 16S (healthy = 0.0336; CRC = 0.0234) data (Fig. S1D and Fig. S1E). ANOSIM analysis indicated that CRC samples were significantly more divergent from one another than healthy samples for both transcriptomic (*P = *0.043) and 16S (*P = *0.002) data (Fig. S1F and Fig. S1G).

10.1128/msphere.00626-22.1 FIG S1 Community diversity of gut microbiota based on meta-transcriptomic sequencing and amplified 16S markers. (A) Spearman’s correlation between 16S rRNA gene and metatranscriptome data sets. (B) PCoA ordination plot of non-CRC- (healthy) and CRC-associated active taxonomy. (C) PCoA ordination plot of non-CRC- and CRC-associated microbial 16S abundance-based taxonomy. (D) The Gini-Simpson α-diversity index (1-Simpson) for each sample’s active taxonomy. (E) The Gini-Simpson α-diversity index (1-Simpson) for each sample’s 16S abundance-based taxonomy. (F) ANOSIM results, based on Bray-Curtis dissimilarity of samples, showing the distribution of ranks of pairwise dissimilarities between non-CRC and CRC-associated active taxonomy, and among non-CRC and CRC-associated active taxonomy. (G) ANOSIM results, showing the distribution of ranks of pairwise dissimilarities between non-CRC and CRC-associated 16S taxonomy and among non-CRC and CRC-associated 16S taxonomy. The plots shows that CRC-associated samples are significantly more dissimilar from one another than the healthy microbiota in both metatranscriptome (*P* = 0.043) and 16S (*P* = 0.002) data sets. Principal coordinate analysis, PCoA. And analysis of similarity, (ANOSIM). Download FIG S1, TIF file, 2.6 MB. Copyright © 2023 Lamaudière et al. 2023 Lamaudière et al. https://creativecommons.org/licenses/by/4.0/ This content is distributed under the terms of the Creative Commons Attribution 4.0 International license.

10.1128/msphere.00626-22.3 TABLE S1 A. Clinical metadata of the participants collected by UHCW. B. PERMANOVA analysis of participant metadata. C. Species that are detected only at the transcriptome or genome level. Download Table S1, XLSX file, 0.02 MB. Copyright © 2023 Lamaudière et al. 2023 Lamaudière et al. https://creativecommons.org/licenses/by/4.0/ This content is distributed under the terms of the Creative Commons Attribution 4.0 International license.

### Members of the gut microbiota display divergent levels of abundance and transcriptional activity in CRC and non-cancerous environments.

Dormancy, an adaptive mechanism adopted by bacteria, is critical to withstand environmental pressures by minimizing metabolic activity (14). In this study, 34 species were identified at the rDNA level with no traces of transcriptional activity, 8 of those were present in the CRC gut and absent in the control group (Table S1C). Bacteroides gallinaceum, Helicobacter canadensis, Corynebacterium durum and, and *Dialister* sp. were among species common to both groups with undetectable expression. Corynebacterium amycolatum, Mesorhizobium plurifarium, Sphingopyxis terrae, and *Promicromonospora* sp. etc. were unique to the CRC gut. All microbes compared at the DNA and RNA levels were present in both databases (15, 16) (including previous taxonomic classifications). Although it is possible that the lack of transcripts from these rare microorganisms may be due to faster mRNA degradation in some species relative to others, we conclude that those organisms are transcriptionally inactive or dead.

**FIG 1:**
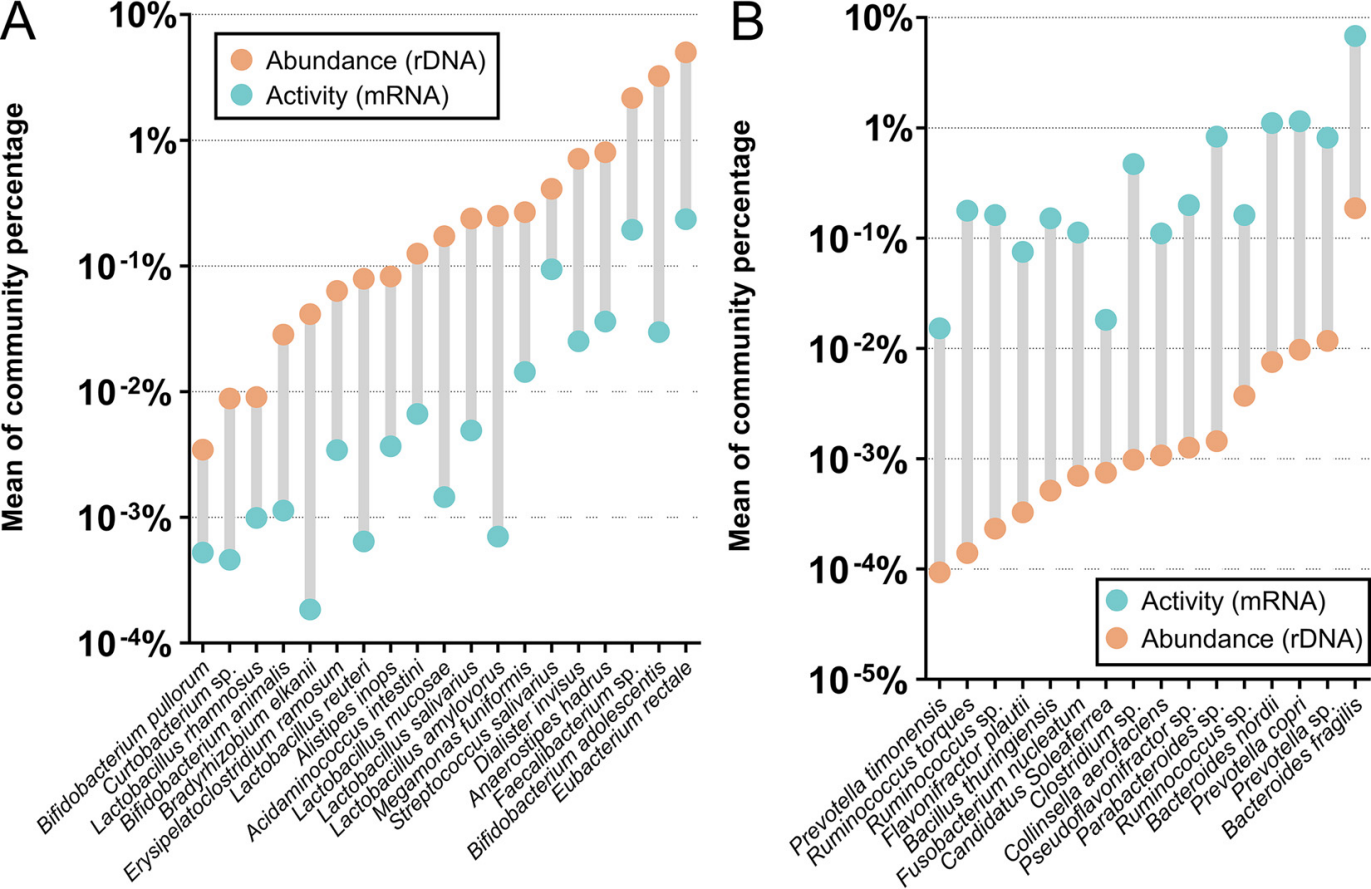
Relative abundance and activity are affected by the CRC gut niche independently. (A) Species abundancethat (level of 16S rDNA) overrepresents their corresponding activity (level of expressed mRNA). (B) Species activity that overrepresents their corresponding abundance. All data presented are a mean percentage of total microbial population (abundance) or transcriptome (activity) across both cohorts.

A further subset of species showed a detectible transcriptome level while their relative abundance was greater by at least an order of magnitude (Fig. 1A), suggesting a dormant-like phenotype. A dormant microbial cell will still have low mRNA levels which can be used for housekeeping or translationally silent (17). The low presence of a dormant species would very likely result in failure to detect its transcripts. For instance, Chlamydia trachomatis was represented in the CRC population more than 430-fold compared to that of their relative activity, but this DNA:RNA ratio was down to 8.3-fold in the control group. Members of this sub-set of microbes, strikingly, includes species that hold significant probiotic potential. *Bifidobacterium* species B. adolescentis, *B. animalis*, and *B. pullorum* have relative abundances 10 to 60-fold greater than their relative activity in both groups (excluding *B. pullorum* in CRC whose activity is greater relative to its abundance). The proportional abundance of B. adolescentis (a bacterium which maintains the gut-brain axis) (18) made up 3.25% of the microbiome in both disease and control groups. However, the proportion of their RNA levels was at least 100-fold less in CRC. This further supports the idea that the CRC environment can regulate activity of microbes without changing their presence. This trend is even more pronounced for several *Lactobacillus* species, e.g., *L. amylovorus*, L. reuteri, and *L. mucosae*. *Bifidobacterium* spp. and *Lactobacillus* spp. share many phenotypic characteristics and are often considered for probiotic administration due to their acid fermenting capabilities (19). However, the realization of their potential (16S level) appears to be limited, thus their likely protective role may be overestimated. Consistently, Eubacterium rectale (inflammation-causing species), Streptococcus salivarius, and *D. invisus* follow this dormant-like pattern. Although roles of certain species may appear significant to health and disease due to their genome abundance, they may be overestimated in their contribution.

Contrarily, we found many transcriptionally active microorganisms in both cohorts that were largely absent (genome level) in at least 1 group (Table S1C). The active species, Bacteroides dorei, *Duodenibacillus massiliensis*, Alistipes shahii, Bacteroides cellulosilyticus, and Acinetobacter baumannii (a clinically relevant multi-drug resistant ESKAPE group opportunistic pathogen) (20) had no detectable rDNA. Pro-inflammatory *A. shahii*, associated with optimal responses to cancer immunotherapy (21), and Acinetobacter junii, linked to nosocomial pneumonia, were also absent at 16S levels.

Additionally, several species were identified with relative activity far greater than their respective representation in the community, indicating ‘over-active’ species relative to population size (Fig. 1B). For instance, Ruminococcus torques, a CRC-associated bacterium (8), displayed the most divergent relationship between relative presence and activity, with an average of ~1.3 × 10^3^-fold difference between the DNA and RNA levels, in both healthy and CRC. Bacteroides nordii, Bacillus thuringiensis, F. nucleatum, Prevotella timonensis, *Megamonas* sp., Prevotella copri, Flavonifractor plautii, B. fragilis, and Collinsella aerofaciens constitute further examples of bacteria, including CRC-associated species, where relative transcriptional activity was disproportionately greater than abundance within the community. Moreover, ecologically under-characterized species within this subset, such as *Candidatus soleaferrea* (22), warrant more detailed functional analysis. This data highlights that genome data is a poor indicator of microbial activity in the community and may lead to the underestimation of certain potentially relevant pathogens roles in CRC.

### Activity of the microbiome in CRC shifts away from beneficial species toward a diverse range of pathogens.

**FIG 2:**
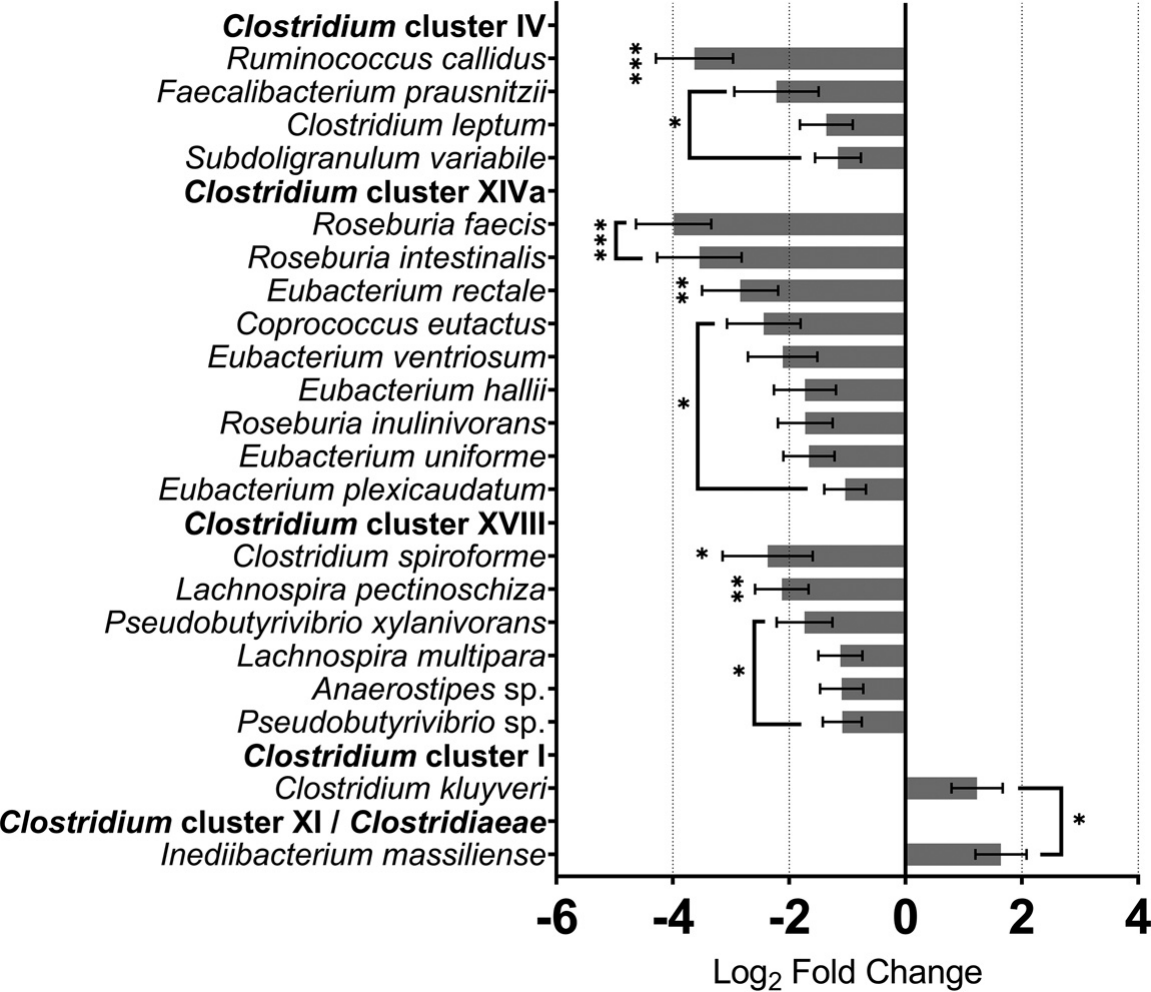
Beneficial butyrate producing species are less active in CRC while some pathogenic butyrate-producers gain activity. Species that possess butyrate-synthesizing genes activity is widely affected in the CRC gut. Beneficial *n*-butyrate producing bacteria belong to *Clostridium* clusters IV, XIVa, and XVIII, where the activity is significantly changed in CRC. Pathogenic *n*-butyrate producing *Clostridium* cluster I, Clostridium kluyveri, and *Clostridium* cluster XI/*Clostridiaceae*, *Inediibacterium massiliense* (91) are also shown, however, with elevated activity in the malignancy. *, *P ≤ *0.05; **, *P ≤ *0.01; ***, *P ≤ *0.001.

A primary role of intestinal microbiota is to provide the host with metabolites critical to the maintenance of gut homeostasis, including short-chain fatty acids (SCFAs), such as *n*-butyrate (23). *n*-Butyrate, the preferred energy source for colonocytes, fortifies the epithelial barrier, and suppresses inflammation (24). *n*-Butyrate is mainly produced by *Clostridium* clusters IV, XIV, and XVI abundances of which have been shown to negatively correlate with CRC (25). We observed, with the exception of Clostridium perfringens (a common cause of food poisoning [26]) (Table 1), Clostridium kluyveri (synthesizing *n*-butyrate from ethanol and lactate), and *Inediibacterium massiliense* (encoding butyrate kinase) whose activities were enhanced, significant loss in activity of 22 major *n*-butyrate producing species (Fig. 2). This is in line with transcription of *n*-butyrate-metabolizing genes/pathways (27). We also found that activity of several *Streptomyces* species (major antibiotic-producing bacteria which control microbial community composition [28]) was diminished in CRC (Table 1). Interestingly, these 3 species (out of 10 identified) contributed almost half (48%) of all *Streptomyces* activity in health with undetectable levels of rDNA, reducing their activity in CRC to 9%. This suggests that specific *Streptomyces* species may be important for the health of the gut.

**TABLE 1:** Opportunistic pathogens that may cause infections in immunocompromised humans and their activity that is regulated in CRC^*a*^

Organism name	Log2 fold change	lfcSE	*Padj*
Upregulated in CRC			
Actinomyces cardiffensis	1.92	0.70	0.0972
Actinomyces dentalis	1.95	0.66	0.0676
Actinomyces graevenitzii	1.90	0.66	0.0773
Actinomyces israelii	1.86	0.62	0.0629
Actinotignum urinale	1.86	0.63	0.0686
Clostridium perfringens	1.84	0.67	0.0980
Dolosigranulum pigrum	1.18	0.43	0.0999
Enterobacter *lignolyticus*	2.47	0.81	0.0565
Gemella sanguinis	2.44	0.67	0.0253
*Gemella* sp.	1.91	0.70	0.0995
Klebsiella aerogenes	2.43	0.82	0.0676
Klebsiella michiganensis	1.95	0.68	0.0827
Klebsiella quasipneumoniae	2.18	0.78	0.0924
Klebsiella variicola	2.74	0.90	0.0565
Mycoplasma pneumoniae	2.83	0.93	0.0588
Parvimonas micra	2.45	0.62	0.0109
Staphylococcus hominis	3.51	0.74	0.0009
Staphylococcus pasteuri	1.62	0.58	0.0924
Staphylococcus saprophyticus	2.25	0.78	0.0761
Streptococcus agalactiae	1.68	0.60	0.0924
Streptococcus cristatus	1.96	0.63	0.0515
Streptococcus dysgalactiae	1.61	0.50	0.0452
Streptococcus gordonii	2.20	0.60	0.0240
Streptococcus infantis	2.40	0.74	0.0447
Streptococcus mitis	2.11	0.63	0.0395
Streptococcus parasanguinis	3.15	0.80	0.0109
Streptococcus pneumoniae	2.11	0.63	0.0393
Streptococcus porci	2.78	0.76	0.0240
Streptococcus pyogenes	1.58	0.58	0.0995
Streptococcus sanguinis	1.45	0.53	0.0975
Vibrio parahaemolyticus	3.71	0.73	0.0003
Downregulated in CRC			
Aggregatibacter aphrophilus	-2.24	0.70	0.0469
Anaerobiospirillum succiniciproducens	-1.33	0.48	0.0975
Campylobacter insulaenigrae	-2.76	0.88	0.0505
Chryseobacterium gleum	-1.80	0.63	0.0768
*Clostridium neonatale*	-1.02	0.33	0.0502
Haemophilus influenzae	-2.25	0.76	0.0660
Haemophilus parainfluenzae	-2.68	0.81	0.0418
Legionella steigerwaltii	-2.27	0.72	0.0502
Prevotella bivia	-2.33	0.83	0.0882
Pseudomonas citronellolis	-3.57	1.05	0.0373
*Sphingomonas* sp.	-1.65	0.52	0.0487
Eubacterium hallii	-1.73	0.53	0.0447
Eubacterium plexicaudatum	-1.04	0.36	0.0763
Eubacterium rectale	-2.84	0.65	0.0029
Eubacterium uniforme	-1.66	0.44	0.0198
Eubacterium ventriosum	-2.11	0.60	0.0281
Streptomyces albus	-3.58	1.07	0.0001
*Streptomyces mangrovisoli*	-3.86	1.16	0.0398
Streptomyces niveiscabiei	-4.12	1.07	0.0146
ESKAPE Pathogens			
Enterococcus faecium	0.15	0.40	0.8466
Staphylococcus aureus	1.63	0.55	0.0674
Klebsiella pneumoniae	1.56	0.63	0.1392
Acinetobacter baumannii	1.45	0.69	0.2145
Pseudomonas aeruginosa	2.71	0.59	0.0014
Enterobacter *lignolyticus*	2.47	0.81	0.0565

a Table displaying the fold change (Log_2_) in activity of species in CRC compared to non-CRC. Transcriptome levels of 3 ESKAPE bacteria, E. faecium, K. pneumoniae, and A. baumannii was unchanged across conditions: lfcSE, log fold change standard error.

**FIG 3:**
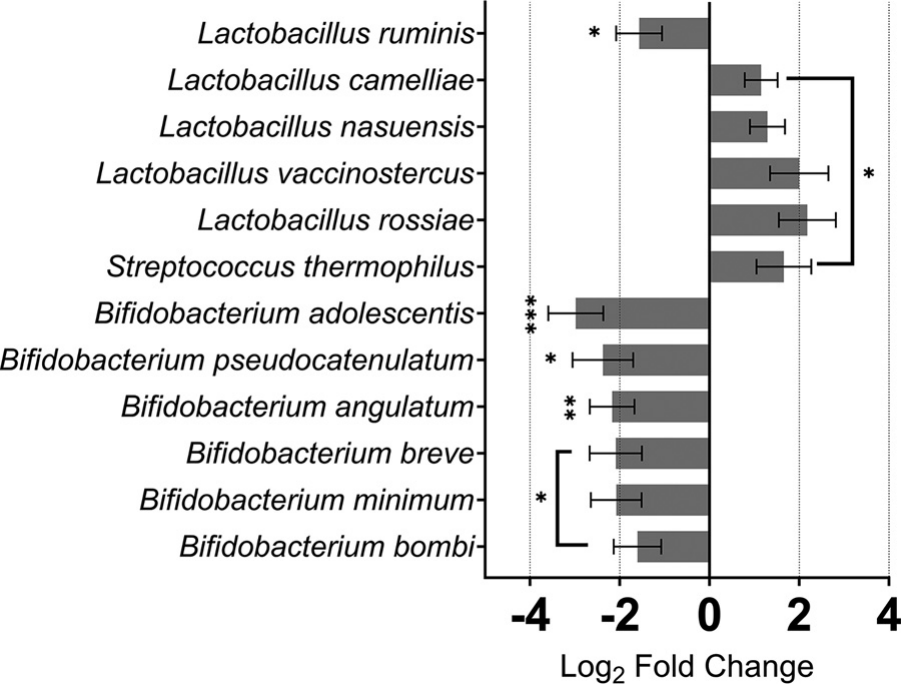
Probiotic genera, including *Lactobacillus* and *Bifidobacterium* show varied alterations in activity in CRC. Species with probiotic capacity from 3 different genera, *Lactobacillus*, *Bifidobacterium*, and Streptococcus exhibit different patterns of expression during malignancy. *Lactobacillus* displayed species-specific regulation of transcription during CRC, *L. ruminis* (beneficial) alongside the *Bifidobacterium* species was less active while other *Lactobacillus* and Streptococcus were more active. *, *P ≤ *0.05; **, *P ≤ *0.01; ***, *P ≤ *0.001.

Metagenome studies have found many lactic acid-producing bacteria, such as *Bifidobacterium* and Streptococcus thermophilus, are underrepresented in CRC (29). We identified that functional activity of six members of the *Bifidobacterium* genus was downregulated in CRC, while activity of a probiotic S. thermophilus was enhanced (Fig. 3). Notably, it has been suggested certain probiotics pose a risk to immunocompromised individuals (30). Interestingly, activity of 4 *Lactobacillus* bacteria was stimulated in cancer, while the activity of another probiotic, Lactobacillus ruminis (31), was reduced. Despite sharing characteristics, namely, lactic acid-producing genes, total activities of these species are differentially regulated under the same CRC conditions.

**FIG 4:**
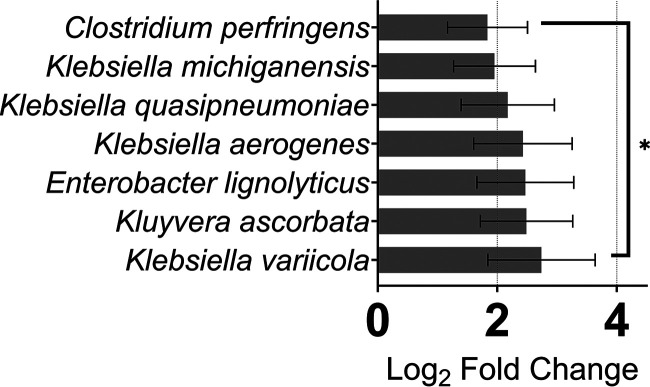
Enterobactiaceae family pathogens have increased activity in the CRC niche. Four genera of the Enterobactiaceae family, *Clostridium*, Klebsiella, Enterobacter, and *Kluyvera* have member species with augmented transcriptional activity during CRC. *, *P ≤ *0.05.

**FIG 5:**
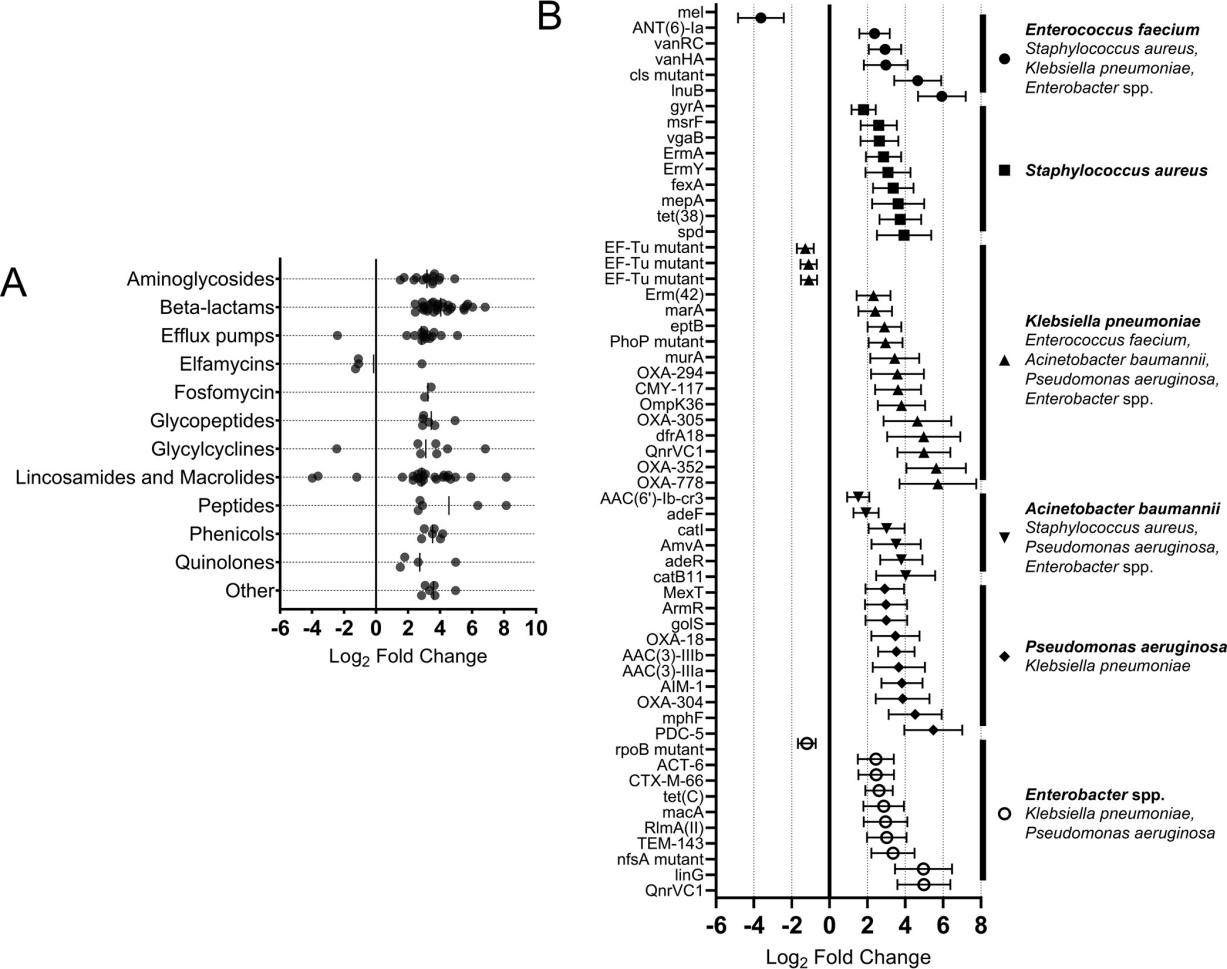
Resistance genes to over 12 families of antibiotics, as well as multidrug resistance, are found differentially expressed in CRC, including by ESKAPE pathogens. (A) CRC microbiota upregulate expression of AB resistance determinants of 12 major families of antibiotics, including aminoglycosides, β-lactams, lincosamides, and macrolides. Other ABs/AB families include synthetic oxazolidinone, rifamycin, streptogramins, pleuromutilin, nitrofuran, isoniazid, and diaminopyrimidine. (B) ESKAPE pathogens of the CRC gut upregulate expression of a gamut of AB resistance determinants. Bold species denote primary expressor of genes, and listed ESKAPE pathogens below bold species can also express genes displayed for that group. All data points plotted are statistically significant (either > 90% or > 95% confidence). Solid black circle, E. faecium; Black square, S. aureus; Upward pointing black triangle, K. pneumoniae; Downward pointing black triangle, A. baumannii; Black diamond, P. aeruginosa; Hollow black circle, Enterobacter spp.

There are a number of pathogens present in the human gut that upon gaining access to the bloodstream may cause life-threatening infections (32), many of which carry multi-drug resistance, such as Enterobacteriaceae (33). We identified members of 3 genera Klebsiella, Enterobacter, and *Kluyvera*, with significantly enhanced activity in CRC (Fig. 4), and, surprisingly activity, of E. coli was not regulated by CRC (Data Set 1, Tab 2), which was confirmed by qRT-PCR (Fig. 5). This aligns, at least in-part, with our functional metatranscriptome analyses that the gut microbiome displayed greater expression of antibiotic resistance determinants in CRC, including β-lactams and efflux pumps (27). The factors responsible for enhancing Enterobacteriaceae activity, such as nutrient availability or colonocyte invasion, are yet to be established.

We identified a set of 31 pathogens that can cause severe invasive infections, particularly in vulnerable immunocompromised individuals (32) with enhanced activity in CRC (Table 1). Among them were Staphylococcus aureus, Pseudomonas aeruginosa, and Mycoplasma pneumoniae that usually in combination cause pneumonia, sinusitis, and diarrhea, in addition to Staphylococcus saprophyticus. Furthermore, 11 Streptococcus species (pathogenic groups A, B, C, and G, e.g., S. pneumoniae, S. pyogenes, S. dysgalactiae, and S. agalactiae) were among these clinically important pathogens.

We also observed repressed activity of 16 pathogens, including Haemophilus sp., Haemophilus parainfluenzae, and *Sphingomonas* sp. (Table 1). Interestingly, this included 5 *Eubacterium* spp., opportunistic pathogens that can produce SCFAs. These 5 species constituted 54% of the *Eubacterium* genus’s transcription in health and only 21% in CRC, suggesting these bacteria may play more health-related, protective roles than pathogenic. ESKAPE pathogens, as recognized by the World Health Organization, are exhausting treatment options as they frequently carry diverse multidrug resistance genes, including extended spectrum β-lactamase, vancomycin, and methicillin resistances (34). All ESKAPE bacteria, including Enterobacter *lignolyticus* were active in both cohorts (Table 1). Enterococcus faecium (0.009% of the total metatranscriptome), Klebsiella pneumoniae (0.136%), and A. baumannii (1.028%) showed no significant difference in their activity. We have identified 2 members of the K. pneumoniae phylogroup, *K. quasipneumoniae* (KpII subgroup [35]) and *K. variicola* (KpIII subgroup) the activities of which were significantly upregulated in CRC. Metatranscriptome levels of S. aureus, P. aeruginosa and *E. lignolyticus* were also significantly higher. These data argue the activity of ESKAPE pathogens is regulated in the CRC gut niche.

### Gut microbiota express antibiotic resistance genes without antibiotic treatment, and transcription of many resistance genes is upregulated by the gut environment.

The surprising data of enhanced AB (antibiotic) resistance determinant expression by the CRC microbiota in the absence of AB treatment of all participants prompted us to investigate differential AB resistome profiles in greater detail (Data Set 1, Tab 4). Analysis of both cohort microbiomes revealed expression of a wide range of AB resistance genes determinants (>1,100 genes), ~10% of which were significantly upregulated in the CRC gut. We found a high level of resistance genes expressed in a number of clinically relevant microorganisms, e.g., S. aureus, C. difficile, Salmonella enterica *serovar Typhimurium*, S. pneumoniae, Neisseria gonorrhoeae and Neisseria meningitidis, Campylobacter jejuni, Helicobacter pylori, E. coli, and Mycobacterium tuberculosis etc, of a wide spectrum of antibiotic classes. Critically, these AB resistance determinants were expressed irrespectively of the health status of the gut. This suggests that the human gut environment supports expression of AB resistances as a part of microbial adaptation. Comparison of expression of AB resistance genes by the CRC and control gut microbiota revealed differential expression of 45 resistance genes (*P < *0.05) with a further 71 genes differentially transcribed with 90% confidence. Observed AB resistance determinants belonged to more than 12 different families (Fig. 5A). Among them, CRC-dependent overexpression of 52 AB resistance genes was observed for all ESKAPE species (Fig. 5B). Interestingly, expression of only 9 AB resistance genes were downregulated in the CRC microbiome, transcription of all other AB resistance determinants was significantly increased in cancer. Strikingly, EF-Tu dependent elfamycin and *rpoB* mediated rifamycin resistances were inhibited. This could be due to a “collateral” effect of acidity of the CRC gut, as low pH affects DNA replication, transcription, and translation (36), hence, expression of AB resistances that target translation and transcription may be affected by downregulation of targeted genes.

One of the striking features of the CRC gut environment is that it differs from the control counterpart with respect to environmental acidic, osmotic, and oxidative pressures (27). We proposed that such factors may, in part, regulate expression of AB resistance genes. Hence, we tested the influence of these environmental factors *in vitro* on microbial expression of AB resistance determinants (Table 2) of CRC and control microbiota grown under aerobic conditions (Data Set 1, Tab 5).

**TABLE 2:** Antibiotic resistance genes for PCR^*a*^

Gene	PCR primers	Amplicon size, nts	Resistance with perfect matches, species of origin	AB resistance	Resistance with sequence variants
*cls*	F_GATCACCGGAAAATTGTTG R_AAGAGACGTTCCAATCCAT	181	Enterococcus faecalis	Daptomycin	N/A
*bla2*	F_ GAAGCAGTTCCTTCGAAC R_ ATCAGCGTGTGCATGTGT	162	Bacillus cereus, Bacillus thuringiensis, Bacillus toyonensis	B1 metallo-β-lactamase, penicillin, cephalosporin, carbapenem	Bacillus anthracis, Vibrio cholerae
*mdtO*	F_ ATGCTCGACTATCCGGAA R_ GCATTTGCGAAATGGCAC	127	Escherichia coli, *Shigella* spp.	Puromycin, acriflavine, nucleoside ABs	*ESKAPE* spp., *Citrobacter* spp., Salmonella spp., Serratia marcescens
*phoP*	F_CTGTCGGTGAATGACCAG R_CGTCGATGGTGTGGCTTT	154	Klebsiella pneumoniae, Escherichia coli	Colistin, Macrolides, Peptide ABs	N/A
*eptB*	F_ CCTTCTTCCTGTTACGTC R_ GATATCGGTGGTCATCAC	161	Klebsiella pneumoniae, Escherichia coli	Peptide ABs	Acinetobacter baumannii, *Citrobacter* spp., Enterobacter spp., Enterococcus faecium, Pseudomonas aeruginosa, Mycobacterium tuberculosis, Serratia marcescens, *Shigella* spp.
*catA*	F_ CAGACCGTTCAGCTGGAT R_ TATCACCAGCTCACCGTC	150	Acinetobacter baumannii, *Citrobacter* spp., Proteus mirabilis, Serratia marcescens, Shigella flexneri, Klebsiella pneumoniae	Chloramphenicol	Alcaligenes faecalis, Chlamydia trachomatis*a,* *Citrobacter* spp., Enterobacter spp., Helicobacter pylori, Neisseria gonorrhoeae, Pseudomonas aeruginosa, Proteus spp., Serratia marcescens, *Shigella* spp.
*bla_CMY_*	F_ GCTGCTGACAGCCTCTTT R_ TGCGTGACTGGGTGGTTA	198	Citrobacter freundii, Providencia rettgeri, Klebsiella pneumoniae, Escherichia coli	CMY-type β-lactamase, cephamycins	N/A
*nfsA*	F_ TCCATTCGCCATTTCACT R_ TAATGCTACTGCACTGCA	109	Escherichia coli, *Shigella* spp.	Nitrofurantoin	Citrobacter freundii, Enterobacter cloacae
*marA*	F_ GGACTGGATCGAGGACAA R_ CTGCGGATGTATTGGCCT	135	Escherichia coli, Shigella flexneri	Multi-drug efflux pump	Klebsiella pneumoniae, *Shigella* spp.
*gadX*	F_ TGTCAAGGGACACGCTTT R_ GATAGTTGCGCAACTTCC	142	Escherichia coli, Salmonella spp., *Shigella* spp.	Penam, fluoroquinolone macrolide ABs	N/A

a Table of primers for AB resistance determinants for gene expression analysis by qRT-PCR in response to the CRC gut environment (predicted from our microbial whole-metatranscriptome sequencing analysis, namely acidic, osmotic and oxidative pressures). Target genes were chosen due to their significant regulation in CRC (resistome analysis via the rgi bwt pipeline [Data Set S1, Tab 4]). Targets represent resistances to a variety of different antibiotic classes (peptides, macrolides, phenicols, β-lactams, nitrofurans) and mechanisms of resistance the major facilitator superfamily (MFS) and resistance-nodulation-cell division superfamily, (RND) efflux pumps) (encoded only by aerobic species). Table includes primer sequences, amplicon sizes, species of origin of the resistance determinant, antibiotic conferred resistance to and other species encoding this determinant. *cls*, cardiolipin synthase (required in membrane synthesis; specific mutations of *cls* in *Enterococcus* confer resistance to daptomycin); *bla2*, Zn dependent β-lactamase type-II with a very broad spectrum of activity; *mdtO*, multidrug (major facilitator superfamily) efflux transporter permease subunit; *phoP*, virulence transcriptional repressor of the *macAB* efflux genes; *eptB*, kdo(2)-lipid A phosphoethanolamine 7''-transferase; *catA*, type A-1 chloramphenicol O-acetyltransferase; *bla_CMY_*, class C β-lactamase; *nfsA*, oxygen-insensitive nitroreductase; *marA*, multidrug resistant efflux pump AcrAB transcriptional activator; *gadX*, acid resistance transcriptional activator which enhances expression of *mdtEF*, RND efflux pump; ESKAPE, six highly pathogenic and antibiotic resistant bacterial species, including *E. faecium*, *S. aureus*, *K. pneumoniae*, *A. baumannii*, *P. aeruginosa* and *Enterobacter* spp. AB, antibiotic.

### H_2_O_2_ represses and enhances expression of AB resistance determinates, depending on the health status of the gut.

**FIG 6:**
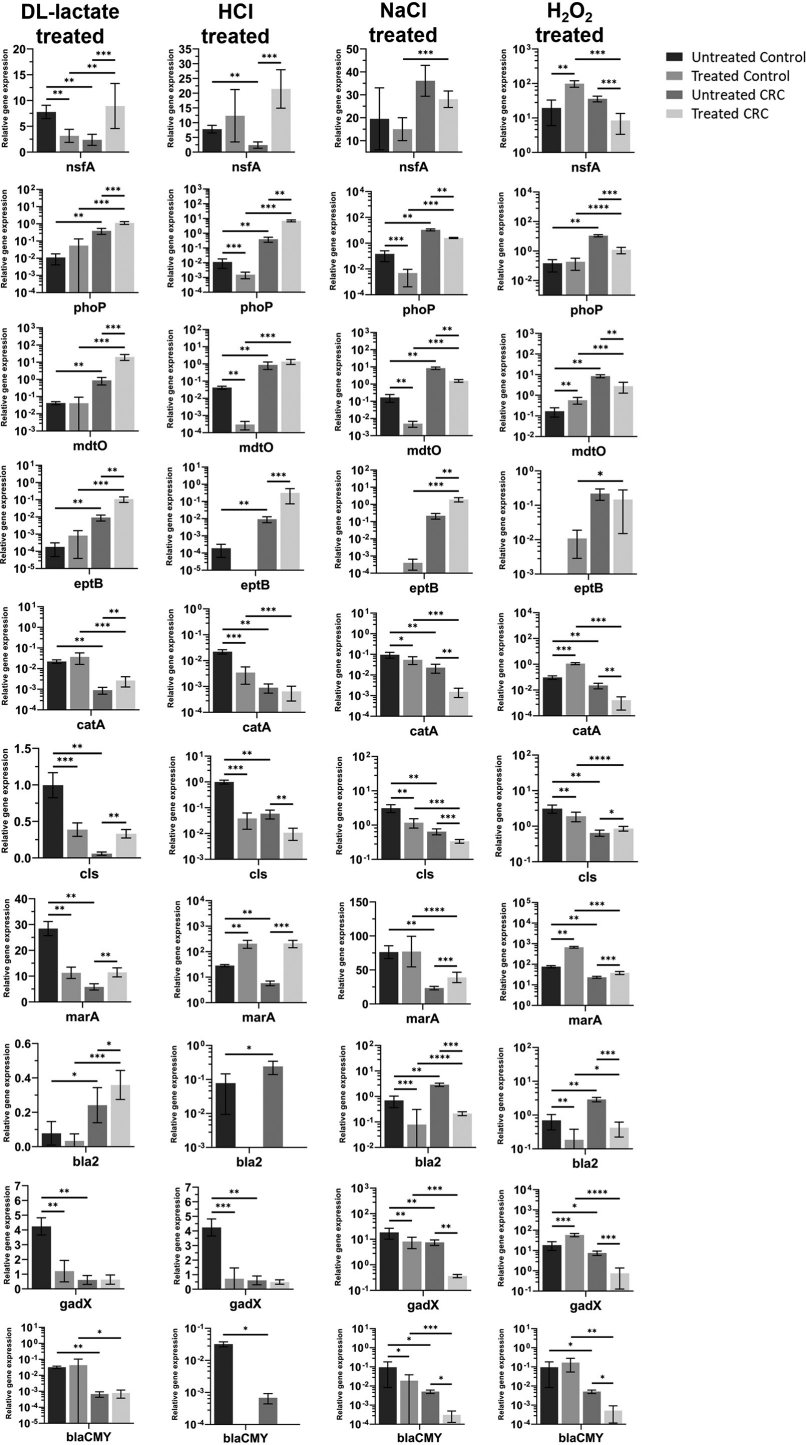
Organic acid pressure induces expression of multidrug resistance determinants. Expression of 10 antibiotic resistance genes was quantified from qRT-PCR, and was conducted following growth of meta-microbiota isolated from both CRC and control cohorts under 4 different CRC-related pressures: DL-lactate (pH 3.5), HCl (pH 3.5), NaCl (5%), and H_2_O_2_ (1.5 mM). Levels of expression are shown in arbitrary relative units. Error bars denote standard deviation (*n* = 9), and asterisks represent statistical significance, *, *P ≤ *0.05; **, *P ≤ *0.01; ***, *P ≤ *0.001; and ****, *P ≤ *0.0001.

Hydrogen peroxide simultaneously upregulated expression of *nsfA*, *mdtO*, *catA*, and *gadX* antibiotic resistance genes of the control microbes while repressing their transcription in the CRC-derived microbes (Fig. 6). Expression of *phoP*, and *bla_CMY_* was also inhibited in the CRC microbes with no difference in its expression in microbes derived from the control samples. Expression of the *bla2* gene was significantly attenuated in both cultures. Metatranscriptomic analyses for these genes showed their transcription was significantly greater in CRC. This suggests that these antibiotic resistance determinants are not regulated by hydrogen peroxide, and this is consistent with our observation that H_2_O_2_ oxidative pressure is not a major feature of CRC, in contrast to the control gut environment (27). Expression of *cls* and *marA* was enhanced in CRC microbes in response to H_2_O_2_. Transcription by E. coli in the control cultures was elevated for *marA* and repressed for *bla2*. Expression of *eptB* was not changed by the CRC E. coli but switched on in the control. This suggests that oxidative pressure is a potential regulator of antibiotic resistance of the gut microbiome in a health-dependent manner.

### Osmotic pressure inhibits AB resistance irrespectively of the health of the host but may activate certain resistance mechanisms.

Osmotic pressure downregulated expression of *cls*, *bla2*, *gadX*, *bla_CMY_*, *mdtO*, *phoP*, and *catA* genes in CRC and control bacteria. Expression of *nsfA* was not affected by NaCl in either culture while expression of *eptB* and *marA* was upregulated by the CRC microbes. The control culture did not change expression of *marA* while expression of *eptB* mirrored the phenotype for oxidative pressure. This data argues that osmotic pressure suppresses antibiotic resistance gene expression in a health-independent manner, while it may activate expression of specific AB resistance genes depending upon the health status of the host.

### Organic and inorganic acids control AB resistance gene expression differently.

Expression of *nsfA*, *eptB*, *marA*, and *phoP* was regulated by acidity in a health-dependent manner, where both acid conditions upregulated the gene expression by CRC aerobes but not in the control, except for *marA* expression in response to inorganic acid. DL-lactate promoted expression of *mdtO*, *catA*, and *bla2* genes in the CRC culture but had no effect of their expression in the control. Transcription of *cls* and *gadX* genes was repressed by lactate in the control cultures and their expression was enhanced and non-changed in the CRC cultures, respectively. Expression of *bla_CMY_* was not regulated by lactate in either culture but inorganic acid supressed its expression in both. Inorganic acid prompted no changes in expression of *gadX*, *mdtO*, or *catA* in the CRC aerobes while attenuated their expression in the control. Furthermore, HCl adjusted acidity significantly downregulated transcription of *cls* and *bla2* genes irrespective of the health status origin of bacteria. Lactate appears to be a major positive regulator of AB resistance gene expression in CRC-derived aerobes and affects resistance gene expression generally in a health-dependent manner. Inorganic acid, in contrast, variably regulated resistance gene expression. HCl-dependent AB resistance gene expression by health-associated aerobes differs to that of lactate; however, there is limited overlap in the patterns of gene expression following exposure to the two acids.

**FIG 7:**
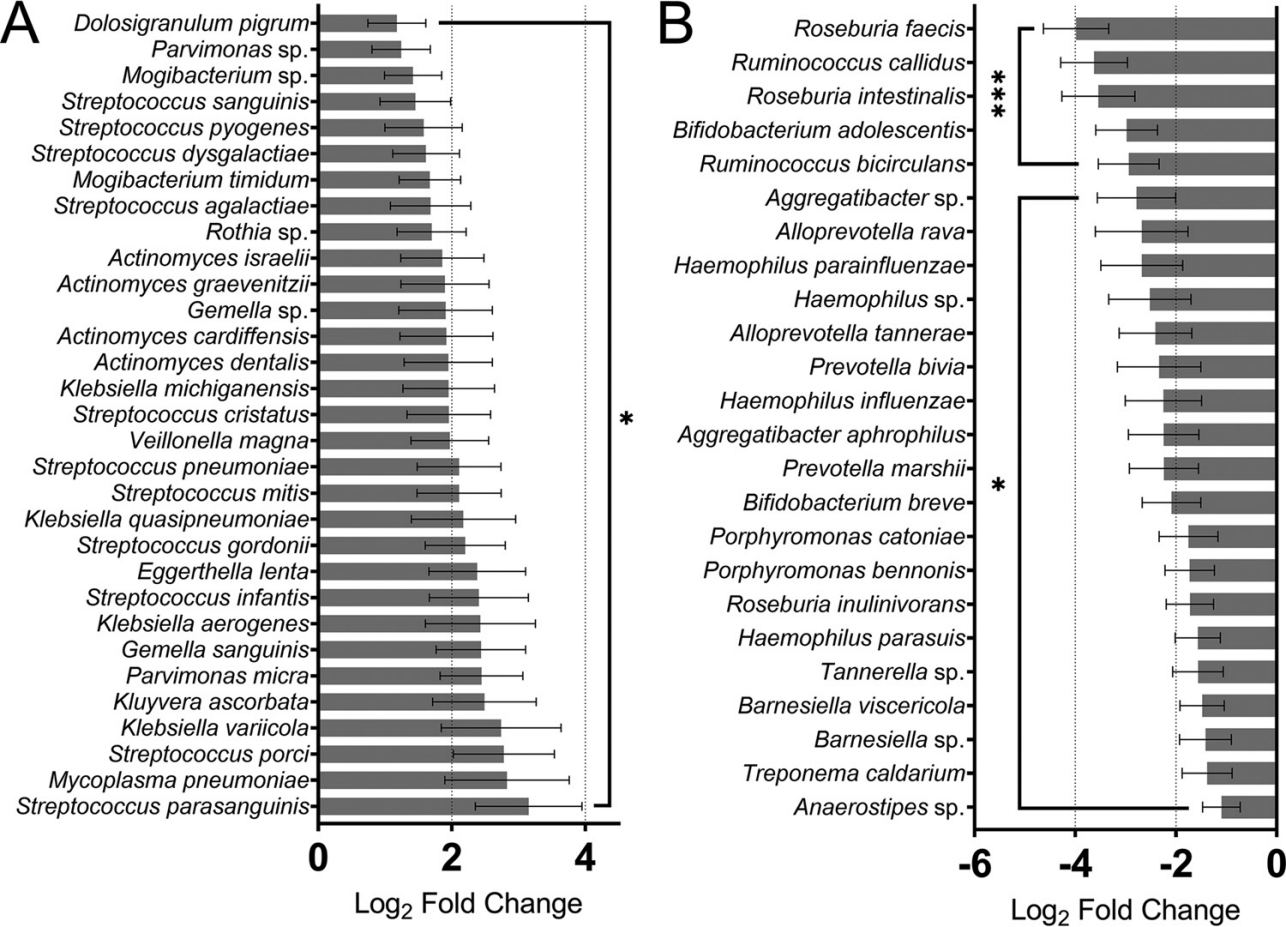
Known early- and late-stage colonizer oral cavity bacteria show CRC-regulated activity. (A) Oral cavity species with elevated activity in the CRC gut. (B) Oral cavity species with diminished activity in the CRC gut. *, *P ≤ *0.05; ***, *P ≤ *0.001.

Several reports have associated CRC with oral cavity microbiota (23), such as Parvimonas micra and Streptococcus spp., suggesting the use of oral bacteria as CRC markers. We observed an extensive collection of oral cavity species which were differentially active across conditions (Fig. 7A). Later colonizers of oral biofilms P. micra, a core CRC-enriched oral pathogen identified through metagenomic analysis (37), and Veillonella magna were among those with elevated activity. Early-stage plaque and biofilm forming species, including *Rothia* sp., *Gemella* sp., 4 *Actinomyces* pathogens, including *A. dentalis*, Streptococcus pyogenes among other Streptococcus species, all which cause periodontal diseases, were more active in the CRC gut. This suggests these pathogens which initiate oral biofilm formation may act with a similar *modus operandi*. Consistent with this, we found that activity of *Anaerostipes* sp. and *Roseburia* are diminished in cancer (Fig. 7B), the increased abundance of which was negatively associated with colonocyte colonization by oral bacteria (38). Furthermore, among bacteria that lost activity in CRC, we observed several oral pathogens which are known for being secondary/later colonizers, such as *Aggregatibacter* sp., pro-inflammatory *Tannerella* sp., *Porphyromonas*, and *Prevotella* (11). Interestingly, their, and Haemophilus spp. genome enrichment in CRC has been reported, further emphasizing the importance of functional analysis. This also suggests that activity of secondary colonizers may be regulated by their ability to adhere to other periodontal pathogens while they may lose out to gut bacteria for secondary colonization.

## DISCUSSION

Through profiling the active CRC microbiome, we identified activity-based marker species of the disease. We also show, for the first time, that expression of antibiotic resistance determinant genes can also be regulated, at least in part by specific gut environmental factors without external antibiotic pressures. Intriguingly, activity of non-cancerous and CRC gut E. coli is fundamentally different, indicative of microbial transcriptional memory.

Changes in the composition of the gut microbiota in CRC have long been reported (37, 39), and common CRC-specific patterns of taxonomy emerged as disease markers. In this work, we show that the abundance and activity of the microbiota in the control and CRC groups do not always correlate, which is in line with metatranscriptome and metagenome data reported for IBD microbiota (10). We found that a number of species exist in a dormant (or dormant-like) state. The opposite was also observed, where several, including previously CRC-linked species, appeared over-active. This argues that activity of specific microorganisms is regulated independently of abundance in CRC. Metatranscriptome levels of many clinically relevant bacteria, such as ESKAPE, Enterobactiaceae, and oral pathogens was significantly elevated in CRC, which is consistent with enhanced β-lactam and vancomycin resistances of this microbiome (27). Responses of aerobes of the CRC and control gut microbiota *in vitro* differed to environmental pressures, particularly to acid pressures. Additionally, multiple commensal (e.g., *n*-butyrate producing) bacteria lose significant activity in the CRC niche.

We identified many microorganisms in one or both groups that exhibited a dormant, dormant-like, and over-active phenotype, which are, potentially, common features of microbial life in the diseased gut (10), by which bacteria can adapt to survive different stresses. As sequencing and analysis methods differ for DNA and RNA data, the possibility of systematic biases between the DNA and RNA data cannot be ruled out. Therefore, we made any direct comparisons with caution and aimed to avoid or reduce biases. For every bacterial species for which we inferred dormancy or over-activity based on a mismatch between its relative abundance in each data set, we ensured that the species was represented in both databases (SILVA SSU rRNA for 16S DNA data, and RefSeq for RNA-seq data), and we analyzed each data type separately when inferring changes in relative abundance between control and CRC. We also, for a subset of species, validated our results using quantitative PCR. The CRC gut features different local oxygen saturation (40) and easier access to colonocytes (27, 41). Such a diseased niche may cause the observed switch from active to latent forms of metabolism. Members of the probiotic *Bifidobacterium* and *Lactobacillus* genera were highly abundant across both cohorts while transcriptome levels were underrepresented by at least 10-fold in comparison. It is noteworthy that CRC patients often present as folate (vitamin B_9_, required for DNA synthesis, methylation, and metabolism) deficient (42), and the diminished activity and dormancy of major *de novo* folate producers, *Bifidobacterium* spp., in the gut niche during cancer may at least, in-part, explain this. The gut environment appears to control activity of probiotic species in a health status-independent manner. Once probiotic administration is ceased, any measurable health benefit quickly diminishes (43), as conditions of the gut likely restricts their activity. Therefore, it would appear manipulating microbial activity could be a more efficient therapeutic approach than probiotic administration to gain any lasting potential health benefits. These findings bring insight into the gut niche dynamically altering, either through amplifying or muting, the metabolic states of different microbes that may have been historically overlooked or well characterized.

We found enhanced activity of Proteobacteria in CRC alongside genome enrichment (Fig. S2C). Over-representation of this phylum in the gut has been widely reported during disease, including CRC (25) and in response to antibiotic treatments (44, 45). However, it has been found that Proteobacteria were under-represented in the microbiota of CRC tissues, particularly E. coli (46, 47), but their activity has not been investigated (48). We report no significant differences in the activity of this bacterium in CRC, confirmed via qRT-PCR. Instead, we observed higher metatranscriptome levels of other Enterobacteriaceae family pathogens, such as Klebsiella, Enterobacter, and *Kluyvera* constituent species, often resistant to antibiotics, and naturally competent to horizontal gene transfer (HT) (49). Proteobacteria do not generally specialize in digesting complex carbohydrates and utilizing fermentation products (50), as they can cross-feed on simple sugars made available through saccharolytic activities of the microbiota. A reduction in carbohydrate and increase in amino acid utilization coincides with an increase in activity of these pathogens, likely augmenting their virulence concurrently (27, 51). These findings suggest a link between carbon source utilization in the CRC gut and enhanced Enterobacteriaceae activity and, potentially, virulence.

Several members of the ESKAPE group, as well as a sub-set of oral pathogens, gain activity in the CRC gut. ESKAPE pathogens are multi-drug resistant carrying bacteria that can acquire said resistance determinants through HT of mobile genetic elements (MGEs) (52). The dissemination of MGEs is predominantly carried out by conjugation, and requires expression of the *tra* operon (53). Activity of 2 ESKAPE pathogens, K. pneumoniae and A. baumannii, was unchanged but their metatranscriptome substantially contributed to the total activity of the microbiome, 0.14% and 1%, correspondently. The observed elevated activity of other ESKAPE pathogens in CRC potentially poses a high risk of infection and the dissemination of MGEs. Acinetobacter, Pseudomonas, and Staphylococcus species can uptake DNA via transformation as well (54). We have observed enhanced conjugative activity of this CRC microbiome (27), including overexpression of *TraM* (DNA transfer) and *TraN* (mating pair stabilization) genes. These findings suggest a potential risk of disseminating resistance determinants and highlight the importance of mapping antibiotic susceptibility patterns of CRC patients to afford appropriate treatment.

Additionally, S. aureus, A. baumannii, and P. aeruginosa can form biofilms, the expression of these determinants is elevated in the CRC microbiome (27), hence facilitating immune evasion, persistence, and antimicrobial resistance (55). We also observed the upregulated transcriptome of several oral cavity pathogens, including primary and secondary tissue colonizers, which consistent with elevated expression of colonization factors by this microbiome (27). We propose that the CRC environment, such as enhanced levels of oxygen and mucus depletion, and altered cross-feeding, triggers significant changes in metabolism of opportunistic oral cavity, ESKAPE, and Enterobacteriaceae pathogens. Therefore, immunocompromised individuals who are subject to recurring infections by these pathogens should be considered vulnerable to neoplasia and offered earlier CRC screening.

The human intestinal microbiota represent a dense microbial population and are natural reservoirs of antibiotic resistant genes (7), and, therefore, the potential for AB gene expression and transfer within such a community is very high (56). RNA-seq analysis has revealed enhanced expression of genes involved in HT and antibiotic resistance to vancomycin and β-lactams alongside an efflux pump in CRC in the absence of AB pressure (27). We found that many AB resistance genes are active in the gut microbiome irrespectively of the health status of the gut with expression of a sub-set of AB resistance determinants significantly upregulated by the CRC microbiota. This leads to 2 important consequences. Firstly, a significant proportion of over-expressed AB resistance genes in CRC were encoded by Enterobacteriaceae. It is known that gut inflammation, a feature of the CRC gut, promotes HT between pathogens and *Enterobacteria* (57), suggesting that CRC microbes are prone to HT, and it would be feasible to expect it is happening in the non-cancerous gut but to a lesser degree. Secondly, expression of numerous AB resistance genes by the gut microbiota strongly argues that the environment of the gut is sufficient to induce AB resistance gene expression. This shows that specific CRC gut (micro)environments may trigger such responses in an AB pressure-independent manner. Enhanced expression of some AB resistance determinants found through RNA-seq in CRC was not determined by a single environmental factor *in vitro*. However, it cannot be ruled out that production of antimicrobials by colonocytes (58) or microbiota may also influence expression of AB resistance genes.

AB resistome data revealed that ESKAPE pathogens display upregulation of AB resistance determinants in CRC, and we confirmed that expression of *catA* by A. baumannii and *phoP* by K. pneumoniae *in vitro* is regulated by environmental factors. Our data shows that multidrug resistant bacteria express their AB resistance genes in response to pressures in a gene specific manner, and often depends upon the health status of the host. It is known that the Salmonella PhoP/PhoQ 2-component system, which is critical for its virulence (59) and confers resistance to colistin, macrolides, and peptide ABs, responds to the host environment and enhances modification of the bacterial envelope, hence reducing membrane permeability (60–63). Exposure to antimicrobial peptides, such as defensins and polymyxin, increases expression of *phoP*/*phoQ* and resistance to antimicrobial peptides (64). Our data shows that nonacid pressures generally inhibit *phoP* expression, but acidity promotes its transcription in a health-dependent manner in CRC-derived K. pneumoniae. This shows that *phoP* transcription can be activated by acidity, which, in turn, may lead to solidification of the cell membrane by enriching the proportion of unsaturated fatty acids (65, 66). It is reasonable to propose that gut bacteria could deploy putative “long-term memory” of acid-dependent membrane solidification.

The altered microbial active taxonomy and community characteristics reported here sets up 2 major questions. Firstly, what are the specific patterns of gene expression of the identified species dominating the microbial transcriptome in both healthy and CRC, and active marker species with dynamic CRC-specific activity. Secondly, what are the CRC-specific environmental factors that drive activity of these species *in vivo*, and the expression and dissemination of genetic elements. Antibiotic resistance appears to be a mechanism that provides the cell with adaptation to environmental pressures in the gut. Thus, it would be of a great importance to investigate combined effects of different pressures on resistance, especially for those that conversely regulate expression of the same AB gene. Effects of colonocyte metabolism, as well as other human tissues on regulation of AB gene expression, should also be a high priority topic in the antibiotic resistance research.

## MATERIALS AND METHODS

### Sample collection.

This study was approved by the University Hospital Coventry and Warwickshire NHS Trust, UK Ethic certificate No: 09/H1211/38. All volunteers provided informed consent prior to participation and for the publication of any research results. Fecal samples from CRC patients and volunteers collected under the auspices of the Famished study at the University Hospital Coventry and Warwickshire NHS Trust (UHCW), UK Ethic certificate No: 09/H1211/38. Ten CRC patients (requiring emergency surgery), and 10 randomized non-CRC fecal samples were collected at UHCW. Samples were immediately frozen in liquid nitrogen upon collection and stored at –80°C. Patient metadata were also collected at UHCW (Table S1A).

### Purification of microbiota from fecal samples.

One gram of fecal sample was resuspended in 25 mL of ice cold, sterile 1X phosphate-buffered Saline (PBS) and centrifuged at 300 × *g* for 10’ at 4°C to pull down the debris. The supernatant was transferred to a new 50 mL falcon tube and centrifuged at 3,000 × *g* for 30’ at 4°C to form a microbial pellet. The supernatant was discarded, and the pellet was resuspended again in 25 mL ice cold 1X PBS for washing, followed by a repetition of the previous centrifugation step. Finally, the washed pellet was resuspended in ice cold 1X PBS containing 20% glycerol in a volume of 6 mL, filtered through Miracloth (Calibochem) to remove any traces of potential aggregates. Aliquots of purified meta-microbiota from each sample of the cohort were pooled to make 2 mL of total CRC and total control meta-microbiota stocks were frozen in liquid nitrogen and stored at –80°C.

### RNA and DNA isolation, and sequencing.

The RNeasy PowerMicrobiome kit (Qiagen) for total RNA extraction was used, following manufacturer protocol. A total of 300 mg of each fecal sample was used. Purified total RNA was stored at –80°C. Total RNA quality and concentration was analyzed using the Agilent Technologies 2100 Bioanalyzer capillary gel electrophoresis system. RNA-seq was carried out by Vertis Biotechnologie AG, Germany, including depletion of rRNA, preparation of cDNA and Illumina NextSeq 500 sequencing (2 × 150 bp paired-end sequencing to produce 2 × 420 M reads). The cDNA inserts were flanked with the following adapter sequences: TruSeq_Sense_primer, i5 Barcode 5′-AATGATACGGCGACCACCGAG ATCTACAC-NNNNNNNN-ACACTCTTTCCCTACACGACGCTCTTCCGATCT-3′ and TruSeq_Antisense_primer, i7 Index 5′-CAAGCAGA AGACGGCATACGAGAT-NNNNNNNN-GTGACTGGAGTTCAGACGTGTGCTCTTCCGATCT-3′. DNA was extracted from 300 mg of fecal sample using DNeasy PowerSoil Pro Kit (Qiagen, 47014), following manufacturer protocol. Blank extractions (300 μL of water) were carried out to assess the quality of the DNA and RNA extraction kits and extractions did not yield any detectable nucleic acids. Total DNA was stored at –80°C. 16S rDNA V3-V4 regions were sequenced by Novogene Co., Ltd on Illumina (NovaSeq 6000 PE150) paired-end platform (100K tags of raw data per sample) to generate 250 bp paired-end raw reads.

### cDNA synthesis.

For qRT-PCR, reverse transcription (RT) of microbial RNA was conducted. Mix^1^ (100 μM d[N_6_] random primer, 25 mM deoxynucleoside triphosphate [dNTP]) of 1.5 μL was added to 4.5 μL of total RNA (0.5 μg/μL), and incubated at 65°C for 5 min to melt the local RNA secondary structure and placed on ice for 2 min. Mix^2^ (5X RT buffer and 100 mM dithiothreitol [DTT]) of 3 μL was added and incubated at 25°C for 2 min for efficient primer annealing. Reverse transcriptase SuperScript II (Invitrogen) 200U (1 μL) was added in a final volume of 10 μL, and samples were incubated at 42°C for 90 min followed by 5 min at 75°C, and stored at –80°C. A 50-fold dilution of cDNA was used for quantitative PCR. The SYBR Green iTaq (Bio-Rad) qRT-PCR system was tested with the 16S rDNA primers for contamination (water used as the template), and DNA contamination of the RNA samples (proportionally to the amount of cDNA used for amplification, diluted RNA samples were added as templates for PCR). No amplification was observed for all the control samples.

### qRT-PCR and qPCR.

To validate taxonomic metatranscriptome and 16S rDNA sequencing data, qRT-PCR and qPCR were conducted, respectively. A total of20 ng/μL genomic DNA (gDNA) or cDNA, 2X i*Taq* Universal SYBR green Supermix (Bio-Rad), species-specific *argS* (Table 3), and universal 16S rRNA gene primers, 1369F-forward and 1492R-reverse (67) at aF final concentration of 0.5 μM were used for taxonomic analysis and normalization, respectively. Hot start for polymerase activation was carried out at 95°C for 2 min, followed by 35 cycles of qRT-PCR using cDNA as a template (15 cycles for qPCR using gDNA as a template) of 15 s at 95°C, 30 s at 56°C and 45 s at 72°C. Melt curves were conducted from 65°C to 95°C with an increment of 0.5°C being held for 5 s. PCR products were visualized by agarose gel electrophoresis, followed by gel purification, TA cloning with pGEM-T Eazy (Promega), and sanger sequencing of 20 randomly selected PCR clones were carried out for all tested genes to validate primer target-specificity of each primer set. Mann-Whitney U tests for qPCR and qRT-PCR (*P*_adj_ < 0.05) were conducted to establish statistically significant differences in gene presence and expression, correspondently, between the CRC and control groups.

**TABLE 3:** Primers used for qRT-PCR to confirm the metatranscriptome analysis

Organism name	Forward primer	Reverse primer	Gene name	Annealing temp
Faecalibacterium prausnitzii	CACCGGCAAACTGCACAC	GGCCGTCAAGACCGACAA	argS	56°C
Enterococcus faecalis	TGCTTTGTTCGTTGCCGAC	CGGGATTTAGCTGCTGCG	argS	56°C
Prevotella copri	GGGCTGACCTCACCAACG	GAAACGGAGGTCGGCAGT	argS	56°C
Fusobacterium nucleatum	TGCTTGGTTGGGACATTGA	AGCACGGCTATGAGCTTC	argS	56°C
Bacteroides fragilis	ACCAACATCTCACGCGCT	GCAAGGGTGAAACTCCCGA	argS	56°C
Escherichia coli	GAGCAGGTGCTGACTCAT	TTCAAGGTCAGCCAGCTC	argS	56°C
Bacterial universal 16S	CGGTGAATACGTTCYCGG	TACGGCTACCTTGTTACGACTT	16S	56°C

### Expression of antibiotic resistance determinants under different growth conditions *in vitro*.

Two acid stress conditions, HCl and DL-lactate, alongside osmotic stress, NaCFl, and oxidative stress (H_2_O_2_) were used as culture treatments before assessment of differential gene expression. A total of 50 μL of purified meta-microbiota from both cohorts, CRC (*n* = 3) and control (*n* = 3) were cultured in 50 mL LB (pH 6.8) at 37°C until stationary phase (pH 8.4 to 8.6) for 24 h at 200 rpm. Then, 100 μL of control and CRC overnight cultures were transferred into 50 mL of fresh LB pH 5.8 (acid adaptation, *n* = 12) and fresh LB pH 6.8 (“untreated,” *n* = 4) for 2 h as standing cultures at 37°C. After 2 h of acid adaptation, the pH of CRC and control cultures were adjusted to pH 3.5 with either HCl (*n* = 6, CRC culture: *n* = 3 and control culture: *n* = 3) or DL-lactate (*n* = 6, CRC culture: *n* = 3 and control culture: *n* = 3). The untreated CRC (*n* = 2), control (*n* = 2) 6.8 pH cultures, and HCl pH 3.5 and DL-lactate pH 3.5 cultures were further incubated at 37°C as standing cultures. After 2 h of incubation, microbes were collected by centrifugation at 3,000 × *g* for 30’ at 4°C. The pellets were washed with 5 mL cold 1X PBS followed by centrifugation using the same conditions as above, and pellets were immediately frozen in liquid nitrogen and stored at –80°C for RNA and DNA extractions. The above overnight cultures were used for NaCl osmotic stress (*n* = 6) and 1.5 mM H_2_O_2_ oxidative stress (*n* = 6), and untreated CRC (*n* = 2) and control (*n* = 2) cultures. Then, 100 μL of the overnight cultures were transferred into 50 mL of fresh LB pH 6.8 adjusted to 5% NaCl, CRC cultures *n* = 3 and control cultures *n* = 3, and oxidative stress, CRC culture *n* = 3 and control culture *n* = 3 at 37°C as standing cultures. After 2 h, the cultures were treated as above for RNA and DNA purification. Microbial gDNA from overnight cultures (CRC and control, 200 μL each) were purified using the DNeasy PowerSoil Pro Kit as described above. Purified gDNA was used for 16S rDNA sequencing by Novogene to establish the microbial composition of the CRC and non-cancerous aerobic cultures. qRT-PCR was used to quantify the level of expression of specific AB genes, using gene specific primers designed for this study (Table 2) and 16S rDNA primers (67) for normalization, as described above.

A more focused analysis of antimicrobial resistance genes was undertaken using the Resistance Gene Identifier (RGI) software (68). The ’rgi bwt’ pipeline was run to map the meta-transcriptome data to the curated Comprehensive Antimicrobial Resistance Database (CARD) (68) v3.1.4 using bwa (69). It is noteworthy that the current CARD is a poor predicter of the AB resistance for P. aeruginosa, and may give a high rate of false-positive predictions in a Global Isolate Data set (70). Read counts per gene were extracted and used to compare conditions (’healthy’ against ’CRC’) using DESeq2 (71) to identify differentially abundant/expressed antimicrobial resistant (AMR)-associated genes.

### 16S rRNA gene data processing and analysis.

Paired-end reads were assigned to samples based on their unique barcodes and trimmed to remove barcode and primer sequences. Read pairs were merged using FLASH v1.2.7 (72). Quality filtering on the raw tags were performed according to QIIME v1.7.0 (73). Sequences were aligned to the SILVA database using UCHIME (74) to detect and remove chimeric sequences (75). Non-chimeric sequences with ≥97% similarity were clustered into operational taxonomic units (OTUs) using UPARSE v7.0.1090 (76). Representative sequences for each output were mapped against the SILVA SSU rRNA database (77, 78) for taxonomic assignment, with an identity threshold of 80%, using QIIME v1.7.0 (73). OTU abundances were normalized to the sample with the fewest sequences and gene copy number.

### Metatranscriptome data processing and analysis.

Raw reads were processed following the steps of the SAMSA2 (15) v2.2.0 pipeline. First, read pairs were trimmed to remove low quality bases using Trimmomatic (79) v0.36, then overlapping read pairs were merged into single sequences using PEAR (80) v0.9.11. Sequences were ‘ribodepleted’ *in silico* using SortMeRNA (81) v2.1 to remove those representing rRNA. The remaining sequences were translated and assigned to a database of 68,433,538 protein sequences from RefSeq (82) with protein names and taxonomic information, using DIAMOND (83, 84) v0.8.38. Sequences assigned to each taxonomic label were aggregated to give raw count data for each taxon, which were used as raw data for further analyses.

### Statistical analyses of microbial diversity.

To compare the transcriptome-based taxonomic data to similar 16S rRNA-marker data, 16S data were re-processed using QIIME2 (85), denoising and merging reads using DADA2 (86), assigning taxonomy using a Naïve Bayes classifier trained on V3-V4 SILVA (87), and counting sequences assigned to each taxon at taxonomic level 7: species. This gave a broadly equivalent count per taxon, at the species level, for both the 16S data and the RNA-seq data, though due to the different types of data, each data set was necessarily processed differently and taxonomic assignment carried out against different databases. As this could result in systematic biases when comparing between DNA and RNA data, each was generally analyzed separately (independently comparing control to CRC for each data type). Where DNA and RNA data were compared (e.g., to infer dormancy), databases were checked to ensure both contained the species in question so that the species could have been identified in both. Per-taxon read counts for transcriptome and 16S data were both normalized (each data type treated separately) using scaling with ranked subsampling using the SRS (88) R package. Normalized counts were used for ordination by PCoA to calculate alpha- and beta-diversity, and to perform ANOSIM and PERMANOVA analyses, all using the VEGAN (89) v2.6-2 R package. For alpha-diversity, the Shannon and the Gini-Simpson diversity (1-Simpson diversity) were calculated for each sample. For beta-diversity, the Bray-Curtis distance was calculated. Bray-Curtis distance was used in PERMANOVA and ANOSIM analyses. PERMANOVA was applied to assess the influence of clinical and lifestyle factors of the study participants on taxonomic distributions. ANOSIM was applied to identify taxonomic dissimilarly between healthy control and CRC samples. For 16S data, the LEfSe (linear discriminant analysis [LDA] effect Size) package was used to test for significant differences of microbial community composition between CRC and control groups. The significance of observed differences in microbial 16S abundance among groups was evaluated by multiple hypothesis-test for sparsely-sampled features and false discovery rate (FDR) through Metastat. *P* values were obtained by permutation test and q-value calculated by Benjamini-Hochberg FDR (90). For RNA-seq data, DESeq2 (71) v1.26.0 was used to test for significant differences in taxonomic abundance using a generalized linear model, Wald T tests, and *q* values calculated with Benjamini-Hochberg FDR (FDR < 0.1).

### Data availability.

All data were submitted to the European Nucleotide Archive (ENA) under the project accession PRJEB53891 (https://www.ebi.ac.uk/ena/browser/view/PRJEB53891). Statistical outputs of data analyses are available as Supplementary tables.

10.1128/msphere.00626-22.2 FIG S2 (A) qPCR confirmation of 16S sequencing analysis and qRT-PCR confirmation of metatranscriptome sequencing analysis. Patterns of abundance for 6 CRC-associated species established by 16S rDNA sequencing confirmed by *argS* targeted qPCT. *F. prausnitzii* and E. coli both show distinctly altered abundance in CRC, with underrepresentation and enrichment, respectively. All data are presented as Log_2_ fold change of abundance between CRC and control cohorts. (B) Transcriptome profiling confirmed through qRT-PCR. Levels of expression and their fold differences across conditions established through metatranscriptome sequencing were confirmed by qRT-PCR for Escherichia coli and *Faecalibacterium prauznitzii*. Both species were selected for having high levels of transcription, where differential activity across conditions was found in the case of *F. prauznitzii*. (C) Samples demonstrated a typical 16S phylogenetic composition of bacterial gut phyla (1), dominated by Bacteroidetes and Firmicutes, and minority phyla Actinobacteria, Proteobacteria, and Verrucomicrobia. Both Proteobacteria and the minor, Spirochaetota, phylum had elevated and diminished proportional abundances in CRC, respectively. Asterisks denote statistically significant differences. SE, standard deviation. Download FIG S2, TIF file, 1.7 MB. Copyright © 2023 Lamaudière et al. 2023 Lamaudière et al. https://creativecommons.org/licenses/by/4.0/ This content is distributed under the terms of the Creative Commons Attribution 4.0 International license.

10.1128/msphere.00626-22.4 DATA SET S1 Tab 1. Raw and processed RNA-sequencing outputs. Tab 2. Active, transcriptome-based, taxonomic, statistical analysis. Tab 3. 16S rRNA gene abundance-based taxonomic, statistical analysis. Tab 4. Targeted transcriptome-based antibiotic, resistome, statistical analysis. Tab 5. Compositional analysis (16S rRNA gene-based abundance) of the aerobic cultures. Download Data Set S1, XLSX file, 0.7 MB. Copyright © 2023 Lamaudière et al. 2023 Lamaudière et al. https://creativecommons.org/licenses/by/4.0/ This content is distributed under the terms of the Creative Commons Attribution 4.0 International license.
