# Supplementary figures and images for: The Colorectal Cancer Gut Environment Regulates Activity of the Microbiome and Promotes the Multidrug Resistant Phenotype of ESKAPE and Other Pathogens
Source: mSphere. 2023 Feb 27;8(2):e00626-22. doi: 10.1128/msphere.00626-22 (PMC10117110; doi:10.1128/msphere.00626-22)

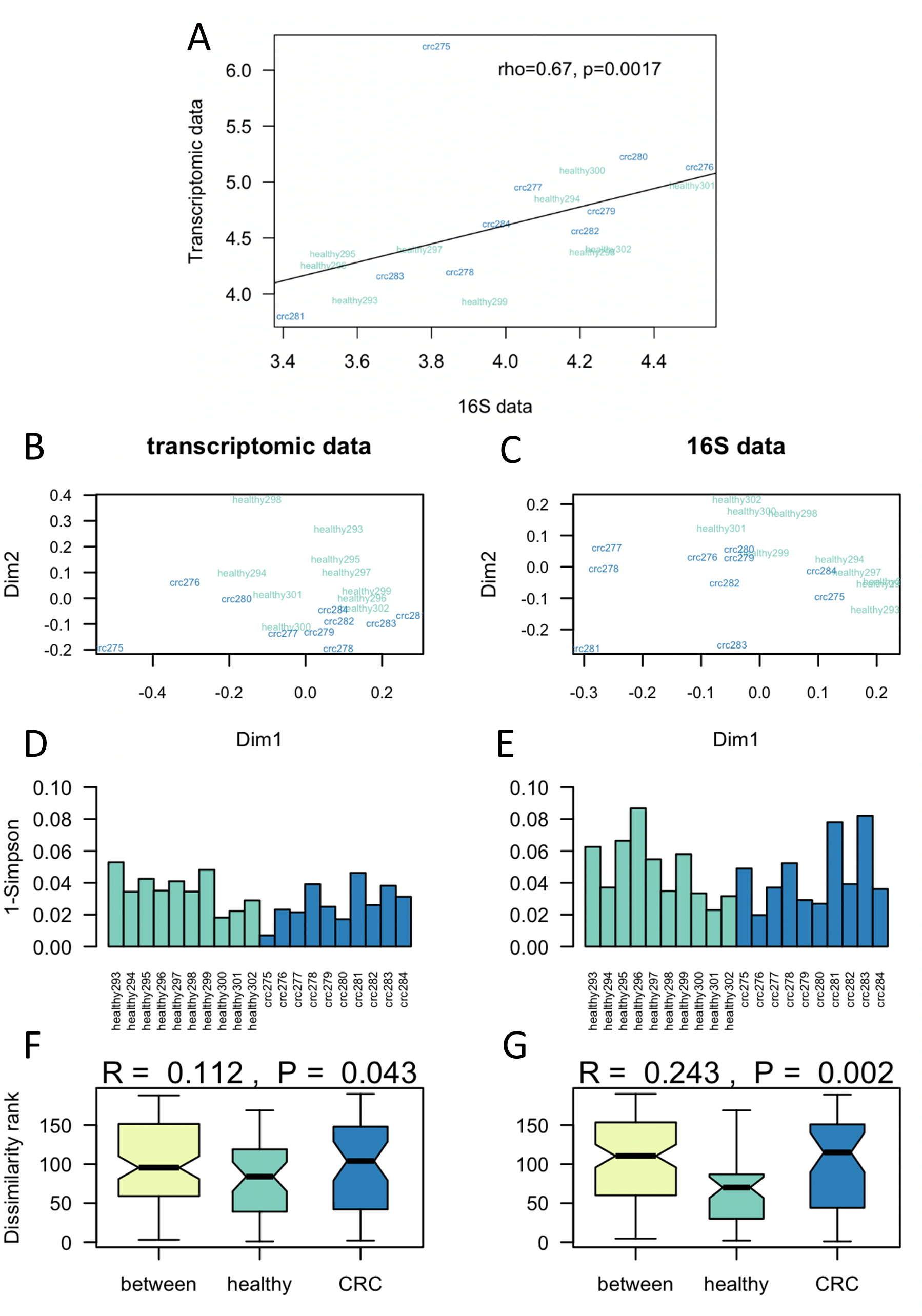

Supplement: FIG S1 [file msphere.00626-22-s0001.tif]

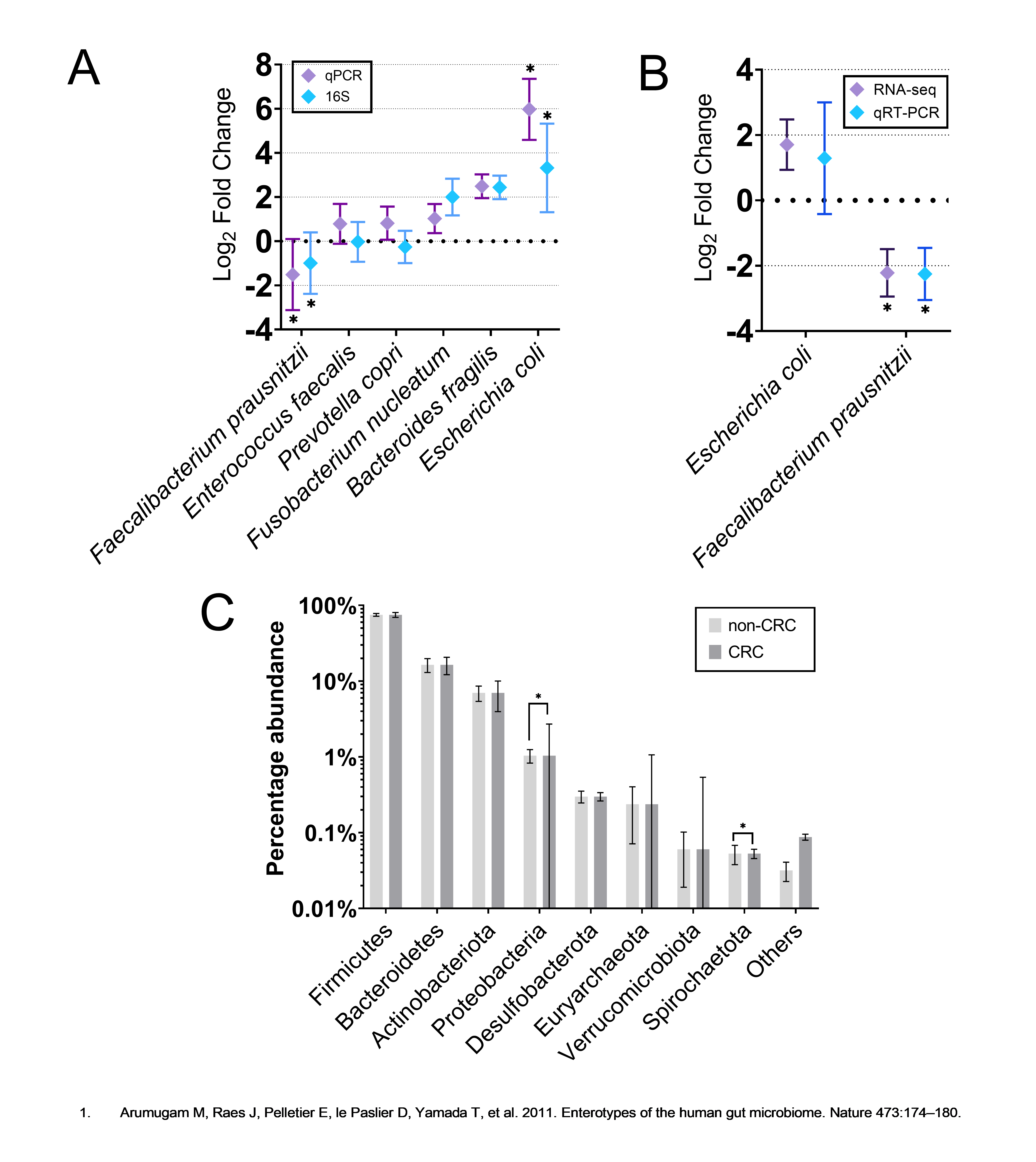

Supplement: FIG S2 [file msphere.00626-22-s0002.tif]
